# Impact of malnutrition and vitamin deficiency in geriatric patients undergoing orthopedic surgery

**DOI:** 10.1080/17453674.2021.1882092

**Published:** 2021-02-04

**Authors:** Matthias Meyer, Franziska Leiss, Felix Greimel, Tobias Renkawitz, Joachim Grifka, Günther Maderbacher, Markus Weber

**Affiliations:** a Department of Orthopedic Surgery, Regensburg University Hospital, Bad Abbach;; b Heidelberg University Orthopedic Hospital, Heidelberg, Germany

## Abstract

Background and purpose — There is growing evidence that hypoproteinemia is an important risk factor for adverse events after surgery. Less is known about the impact of vitamin deficiency on postoperative outcome. Therefore we evaluated the prevalence and impact of malnutrition and vitamin deficiency in geriatric patients undergoing elective orthopedic surgery.

Patients and methods — In a retrospective analysis of 599 geriatric patients who had undergone elective orthopedic surgery in 2018 and 2019, hypoproteinemia, and deficiency of vitamin D, vitamin B12, and folate were assessed. Reoperation rates, readmission rates, complication rates, and transfusion rates were compared between malnourished patients and patients with normal parameters. Multivariable logistic regression models were used to assess the relationship between malnutrition and postoperative adverse events, controlling for confounding factors such as age, sex, diabetes mellitus, and frailty.

Results — Patients with malnutrition showed a higher rate of reoperation (13% vs. 5.5%; p = 0.01) and exhibited more wound-healing disorders (7.4% vs. 1.3%, p = 0.001) as well as Clavien–Dindo IV° complications (7.4% vs. 2.4%; p = 0.03). Deficiency of vitamin D led to a higher rate of falls (8.4% vs. 2.9%, p = 0.006). Deficiency of vitamin B12 and folate did not affect postoperative adverse events. Although correlated to frailty (p = 0.004), multivariable regression analysis identified malnutrition as independent risk factor for reoperation (OR 2.6, 95% CI 1.1–6.2) and wound healing disorders (OR 7.1, CI 1.9–26).

Interpretation — Malnutrition is common among geriatric patients undergoing elective orthopedic surgery and represents an independent risk factor for postoperative adverse events.

Malnutrition is an important risk factor for postoperative complications in orthopedic surgery (Bohl et al. 2016, Kamath et al. 2016). Previous studies found up to 50% of patients to be at risk of malnutrition (Cross et al. 2014). As malnutrition also increases healthcare costs for orthopedic surgery, public and private payers’ growing focus on bundled payment and Value Based Payment models may promote thorough preoperative nutritional screening for economic aspects also (Bala et al. 2020).

Malnutrition is considered as a modifiable risk factor, which is defined as a medical condition that can be altered positively prior to surgery. A recently published prospective study showed that a preoperative nutritional intervention led to improved outcome in malnourished patients undergoing arthroplasty (Schroer et al. 2019). Researching into malnutrition, previous studies mainly focused on the impact of hypoproteinemia on outcome after orthopedic surgery (Cross et al. 2014, Bohl et al. 2016, Kamath et al. 2016). In contrast, less is known about the effects of vitamins and minerals. Epidemiological studies revealed a high prevalence of vitamin D, vitamin B12, and folate deficiency, especially in the elderly (Laird et al. 2018, Sempos et al. 2018). The role of vitamin D deficiency in the context of orthopedic surgery is debated (Maier et al. 2016, Shin et al. 2017, Hegde et al. 2018).

We evaluated the prevalence of malnutrition in a cohort of 599 geriatric patients undergoing elective orthopedic surgery at a high-volume university center. Furthermore, we investigated whether malnutrition, in the form of hypoproteinemia, and deficiency of certain vitamins affected the rate of adverse events after operation.

## Patients and methods

### Study design and study population

This retrospective analysis was based on a database derived from the department’s joint registry and the hospital information system.

As part of the establishment of an orthogeriatric department, all geriatric patients undergoing elective orthopedic surgery had serum levels for total protein, vitamin D, vitamin B12, and folate measured as part of their preoperative blood investigations. Geriatric patients were defined as aged above 65 years with typical geriatric comorbidity or aged above 80 years (Sieber 2007). All patients with complete preoperative laboratory findings and postoperative medical records were included. All operations took place at the Department of Orthopedic Surgery of the University Hospital Regensburg, Germany between January 2018 and December 2019.

Reoperation within 90 days after surgery, readmission within 90 days, complications, and transfusion were defined as endpoints of the study. Complications were categorized into surgical (wound healing disorder, iatrogenic fracture, mechanical complications), internal (myocardial infarction, acute heart failure, cardiac arrhythmias, pneumonia, renal failure) and other complications (deep vein thrombosis, pulmonary embolism, fall, delirium). Furthermore, complications were categorized according to the Clavien–Dindo classification (Dindo et al. 2004). This classification system ranks complications into 5 grades, based on the therapy used for correction. Any deviation from the normal postoperative course without the need for pharmacological treatment or surgical, endoscopic, and radiological intervention represents a Grade I complication. Grade II complications require specific pharmacological treatment, whereas Grade III complications result in surgical, endoscopic, or radiological intervention. Grade IV complications are defined as life-threatening events requiring intensive care management. Grade V represents the death of a patient.

### Definitions of malnutrition and vitamin deficiencies

Malnutrition was defined as total serum protein < 6.0 g/dL (Zhang et al. 2017). In a meta-analysis of biomarkers associated with malnutrition, total serum protein was found to be a useful marker of adult malnutrition (Zhang et al. 2017). Although albumin is the most often used marker for diagnosis of malnutrition, total serum protein performs equally well and is considered less sensitive to acute stressors, which may be useful in perioperative settings (Zhang et al. 2017).

According to the recommendations of the First International Conference on Controversies in Vitamin D (Pisa, Italy, 2017), vitamin D deficiency was defined as serum 25-OH-D level < 20 ng/mL (Sempos et al. 2018). According to Shin et al. (2017) a serum 25-OH-D level < 12 ng/mL was defined as severe deficiency. A serum cobalamin level < 200 ng/L was defined as Vitamin B12 deficiency, whereas a level of 200–300 ng/L was defined as insufficiency (Hunt et al. 2014). Folate deficiency was defined as serum folate level < 2 ng/mL. A serum folate level between 2 ng/mL and 4 ng/mL was defined as insufficiency (Green and Datta Mitra 2017).

### Assessment of frailty

The Hospital Frailty Risk Score (HFRS) was developed in order to provide hospitals with a frailty screening tool derived from routinely collected administrative data. As part of a complex statistical analysis of a geriatric patient cohort, Gilbert et al. (2018) could identify 109 ICD-10 codes characteristic of frailty. Dependent on how strong each ICD-10 code correlated with frailty, different points were awarded to each code and summed to a maximum possible score of 173 points (Gilbert et al. 2018). In a large validation cohort, the HFRS showed fair agreement with common frailty scales (i.e., Fried Phenotype, Rockwood’s Frailty index) and could identify patients at risk of higher 30-day mortality, prolonged length of stay, and readmission (Gilbert et al. 2018). In a previous study the authors found that the HFRS also predicts adverse events in primary total hip and knee arthroplasty (Meyer et al. 2020). The HFRS was calculated retrospectively for each patient based on the available ICD-10 codes that were entered for the time of admission. According to literature frailty was defined as HFRS above or equal to 5 (Gilbert et al. 2018).

### Data collection

Laboratory findings were extracted from the hospital information system (ORBIS; Agfa Healthcare, Mortsel, Belgium). Further available data from our clinical information system were age, sex, length of stay, operative procedure, transfusion, transfer to intensive care unit, reoperation, readmission, and complications, as well as principal and secondary diagnoses at the time of hospitalization including corresponding ICD-10 codes. Diagnostic codes had been entered by professional clinical coders and were double-checked by physicians using information gathered from patients’ medical records.

### Statistics

Continuous data are presented as mean (SD). Group comparisons were performed by 2-sided t-tests. Absolute and relative frequencies were given for categorical data and compared between groups by chi-square tests. All hypotheses in the study were tested on 5% significance level.

**Figure 1.**
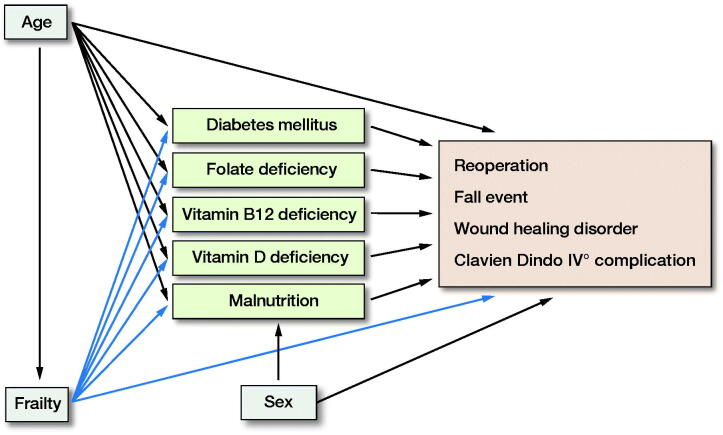
Assumed cause–effect interactions.

Multivariable logistic regression analyses were performed to assess whether malnutrition or deficiency of vitamins is a significant predictor of complications and prolonged length of stay while controlling for other variables known to be associated with adverse surgical outcomes such as age, sex, and frailty (Weber et al. 2018, Meyer et al. 2020). Adjustment of covariates was performed based on considerations regarding cause–effect. The 6-step approach was used to prevent adjustment bias (Shrier and Platt 2008). The assumed cause–effect relation is shown in the appendix (Figure 1, see Supplementary data). IBM SPSS Statistics 22 (IBM Corp, Armonk, NY, USA) was used for analysis.

### Ethics, funding, data sharing, and potential conflicts of interest

The study was approved by the Ethics Committee of the University Hospital Regensburg, Germany (20-1821-104). For this study no funding was received. The data that support the findings of this study is given within the article. Further data can be requested from the corresponding author. The authors have no conflicts of interest to declare that are relevant to the content of this article.

## Results

**Figure 2.**
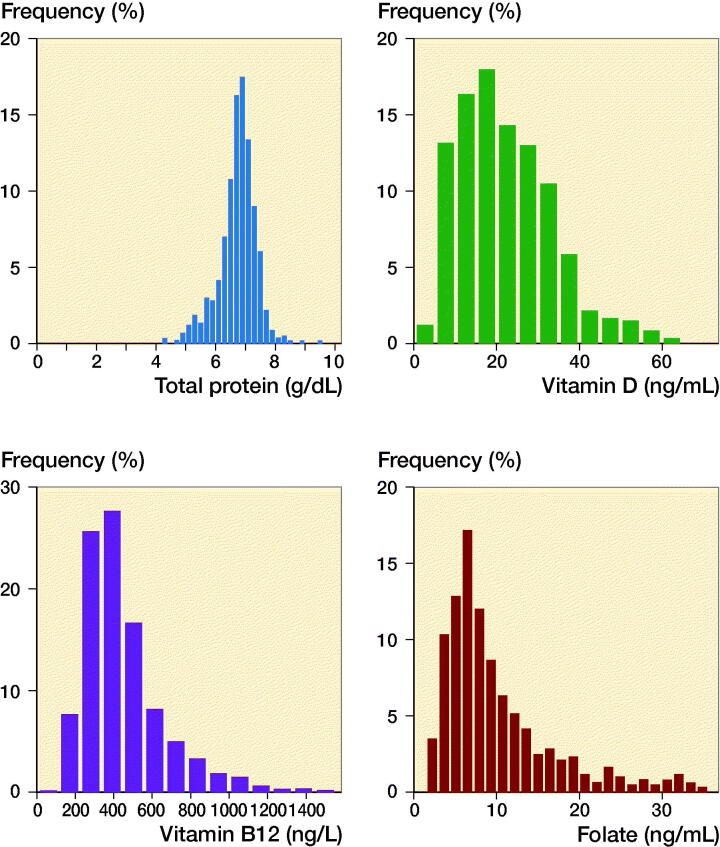
Distribution of total protein serum levels (a), vitamin D serum levels (b), vitamin B12 serum levels (c), and folate serum levels (d) in the study group.

599 geriatric patients underwent orthopedic surgery during the study period (Table 1 and Figure 2). The rate of malnutrition was 11% (68/599). Patients with insufficiency or deficiency of vitamin B12 and folate, respectively, were pooled for further statistical analysis (Table 1).

**Table 1.** Characteristics of the study group (n = 599), frequencies of nutrient deficiencies, and distribution of orthopedic procedure in the study group. Values are frequency (%) unless otherwise specified

Factor	n (%)/mean (SD)
Age (SD)	77 (5)
Sex (women)	387 (65)
Comorbidities	
Diabetes mellitus	101 (15)
Frailty	256 (43)
Nutrient deficiencies	
Malnutrition	68 (11)
Vitamin D deficiency	174 (29)
Severe Vitamin D deficiency	119 (20)
Vitamin B12 insufficiency	118 (20)
Vitamin B12 deficiency	28 (4.7)
Folate insufficiency	60 (10)
Folate deficiency	6 (1.0)
Orthopedic procedure	
Total hip arthroplasty	219 (37)
Total knee arthroplasty	171 (29)
Spine surgery	72 (12)
Foot and ankle surgery	45 (7.5)
Revision total knee arthroplasty	24 (4.0)
Revision total hip arthroplasty	23 (3.8)
Knee surgery	19 (3.2)
Shoulder and elbow surgery	11 (1.8)
Hand surgery	7 (1.2)
Total shoulder arthroplasty	4 (0.7)
Pelvic surgery	4 (0.7)

**Figure 3.**
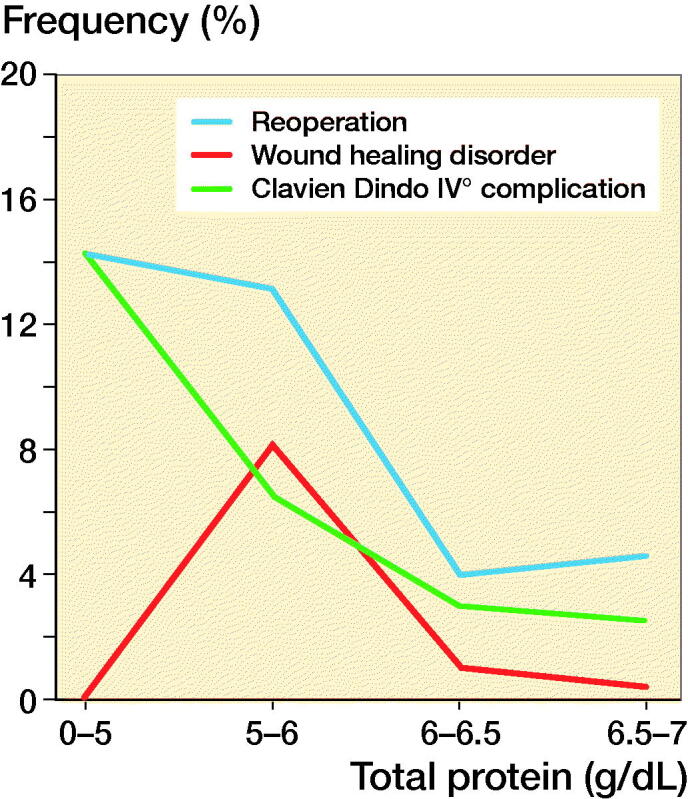
In patients undergoing orthopedic surgery, the frequency of reoperation, wound healing disorders, and Clavien–Dindo IV° complications decreased as total protein serum levels increased.

The malnourished cohort showed a higher reoperation rate (13% vs. 5.5%; p = 0.01) and exhibited more Clavien–Dindo IV° complications (7.4% vs. 2.4%; p = 0.03) than the cohort with normal parameters (Figure 3). Malnourished patients also had higher rates for readmission (8.8% vs. 5.5%; p = 0.3) as well as transfusion (8% vs. 4%; p = 0.09) and exhibited more surgical (16% vs. 9.0%; p = 0.06), other (12% vs. 7.6%; p = 0.2) and internal complications (7.4% vs. 3.6%; p = 0.1) than normally nourished patients, but the increase was not statistically significant. However, as a part of surgical complications, the frequency of wound-healing disorders was significantly higher in patients with malnutrition (7.4% vs. 1.3%; p = 0.001; Table 2 and Figure 3).

**Table 2.** Adverse events after orthopedic surgery according to nutritional status. Values are frequency (%)

Adverse events	Total protein		
	< 6.0 g/dL	≥ 6.0 g/dL	p-value
	n = 68	n = 531	
Reoperation	9 (13)	29 (5.5)	0.01
Readmission	6 (9)	29 (5.5)	0.3
Surgical complications	11 (16)	48 (9.0)	0.06
Wound healing disorder	5 (7)	7 (1.3)	0.001
Iatrogenic fracture	3 (4)	7 (1.3)	0.06
Mechanical complication	3 (4)	34 (6.4)	0.5
Internal complications	5 (7)	19 (3.6)	0.1
Other complications	8 (12)	40 (7.5)	0.2
Clavien–Dindo IV°	5 (7)	13 (2.4)	0.03
Transfusion	6 (9)	22 (4.1)	0.09

Patients with severe vitamin D deficiency exhibited more other complications (13% vs. 6.7%; p = 0.02) than patients with higher serum levels. This was due to a higher rate of falls (8.4% vs. 2.9%; p = 0.006) in the severely vitamin D deficient cohort. Severely vitamin D deficient patients also showed a higher rate of delirium (5.9% vs. 3.1%; p = 0.2; Table 3) than patients with higher serum levels, but the increase was statistically not significant.

**Table 3.** Adverse events after orthopedic surgery according to vitamin D status. Values are frequency (%)

Adverse events	Vitamin D		
	< 12 ng/mL	≥ 12 ng/mLl	p-value
	n = 119	n = 480	
Reoperation	11 (9)	27 (5.6)	0.1
Readmission	9 (8)	26 (5.4)	0.1
Surgical complications	14 (12)	45 (9.4)	0.4
Internal complications	6 (5)	18 (3.8)	0.5
Other complications	16 (13)	32 (6.7)	0.02
Deep vein thrombosis	1 (1)	2 (0.4)	0.6
Pulmonary embolism	1 (1)	1 (0.2)	0.3
Fall	10 (8)	14 (2.9)	0.006
Delirium	7 (6)	15 (3.1)	0.2
Clavien–Dindo IV°	6 (5)	12 (2.5)	0.1
Transfusion	5 (4)	23 (4.8)	0.8

Insufficiency or deficiency of Vitamin B12 or folate, respectively, neither affected reoperation rate nor rates of readmission, complications, and transfusion (Table 4).

**Table 4.** Adverse events after orthopedic surgery according to vitamin B12 and folate status. Values are frequency (%)

Adverse events	Vitamin B12			Folate		
	< 300 ng/mL	≥ 300 ng/mL	p-value	< 4 ng/mL	≥ 4 ng/mL	p-value
	n = 146	n = 453		n = 66	n = 533	
Reoperation	7 (5)	31 (6.8)	0.4	5 (8)	33 (6.2)	0.6
Readmission	9 (6)	26 (5.7)	0.8	5 (8)	30 (5.6)	0.5
Surgical complications	15 (10)	44 (9.7)	0.8	6 (9)	53 (9.9)	0.8
Internal complications	7 (5)	17 (3.8)	0.6	2 (3)	22 (4.1)	0.7
Other complications	11 (8)	37 (8.2)	0.8	6 (9)	42 (7.9)	0.7
Clavien–Dindo IV°	3 (2)	15 (3.3)	0.4	4 (6)	14 (2.6)	0.1
Transfusion	6 (4)	22 (4.9)	0.7	6 (9)	22 (4.1)	0.08

The proportions of patients with frailty (59% vs. 40%; p = 0.004) was higher in the malnourished cohort. Despite its correlation to frailty, multivariable regression analysis identified malnutrition as an independent risk factor for reoperation (OR 2.6; 95% CI 1.1–6.2) and wound healing disorders (OR 7.1; CI 1.9–26). Moreover, severe vitamin D deficiency was identified as independent risk factor for falls (OR 3.1; CI 1.3–7.6; Table 5).

**Table 5.** Multivariable analysis for total effect of malnutrition on reoperations, wound healing disorders, falls, and Clavien–Dindo IV° complications after orthopedic surgery

Variable	OR (95% CI)	p-value
Reoperation		
Malnutrition	2.6 (1.1–6.2)	0.04
Severe Vitamin D deficiency	1.4 (0.62–3.0)	0.5
Folate deficiency	0.84 (0.29–2.4)	0.8
Vitamin B12 deficiency	0.58 (0.24–1.4)	0.2
Diabetes mellitus	1.5 (0.67–3.4)	0.3
Frailty	1.1 (1.0–1.2)	0.05
Age	0.99 (0.93–1.1)	0.8
Sex (male)	1.8 (0.89–3.5)	0.1
Wound healing disorder		
Malnutrition	7.1 (1.9–26)	0.003
Severe Vitamin D deficiency	1.2 (0.31–4.7)	0.8
Folate deficiency	0.31 (0.03–2.9)	0.3
Vitamin B12 deficiency	0.19 (0.02–1.6)	0.1
Diabetes mellitus	3.2 (0.87–12)	0.08
Frailty	1.1 (0.95–1.2)	0.2
Age	1.0 (0.89–1.1)	1.0
Sex (male)	0.73 (0.19–2.9)	0.7
Falls		
Malnutrition	0.58 (0.15–2.3)	0.4
Severe Vitamin D deficiency	3.1 (1.3–7.6)	0.01
Folate deficiency	1.1 (0.34–3.8)	0.8
Vitamin B12 deficiency	0.92 (0.32–2.7)	0.9
Diabetes mellitus	0.34 (0.08–1.5)	0.2
Frailty	1.1 (1.0–1.2)	0.009
Age	1.0 (0.94–1.1)	0.5
Sex (male)	0.48 (0.17–1.4)	0.2
Clavien–Dindo IV° complication		
Malnutrition	2.7 (0.80–9.0)	0.1
Severe Vitamin D deficiency	1.5 (0.48–4.4)	0.5
Folate deficiency	1.8 (0.49–6.3)	0.4
Vitamin B12 deficiency	0.46 (0.13–1.7)	0.2
Diabetes mellitus	2.8 (0.97–7.8)	0.06
Frailty	1.0 (0.91–1.2)	0.6
Age	1.0 (0.93–1.1)	0.6
Sex (male)	1.8 (0.67–4.8)	0.2

OR = odds ratio. CI = confidence interval. For assumed cause–effect relation please see Supplementary data.

## Discussion

The proportion of malnourished patients in our cohort was 11%. Previous studies found 4–50% of patients undergoing elective orthopedic surgery to be malnourished (Cross et al. 2014, Bohl et al. 2016, Kamath et al. 2016, Schroer et al. 2019). The majority of these studies define malnutrition as a serum albumin < 3.5 g/dL. In the context of malnutrition, serum albumin serves as standard serological biomarker. In their review of blood biomarkers associated with malnutrition, Zhang et al. (2017) promoted the use of total protein, as it is considered less sensitive to acute disease stress. As a consequence, total protein might perform better for diagnosis of malnutrition in geriatric patients, who are prone to chronic inflammation.

Regarding vitamins, we found vitamin D deficiency in 49% of patients, with 20% of patients being severely vitamin D deficient. In a retrospective analysis of over 1,000 patients aged over 70 years, who underwent hip or knee arthroplasty, Maier et al. (2016) found vitamin D deficiency in 60% of cases. In contrast Hegde et al. (2018) reported vitamin D deficiency in only 13% of patients undergoing knee arthroplasty. Beyond selection bias, the variable proportion of vitamin D deficiency in patients undergoing joint replacement is likely due to endemic reasons as vitamin D level is affected by sun exposure, dietary supplementation, and genetic differences (Tran et al. 2017).

Insufficiency or deficiency of vitamin B12 and folate was found in 24% and 11% of patients, respectively. To the best of our knowledge, this is the first study to evaluate deficiency of vitamin B12 and folate in the context of orthopedic surgery. In a population-based cross-sectional analysis of 3,511 people aged 65 years or older, Clarke et al. (2004) found 5% of people aged 65–74 years and 10% of people aged 75 years or older to be deficient in vitamin B12 or folate.

We found a statistically significant correlation between malnutrition and postoperative adverse events. Malnourished patients were 6 times more likely to suffer from wound-healing disorders and showed a 3-fold increased rate of complications requiring ICU management. The risk of reoperation and transfusion was more than 2-fold increased for patients with malnutrition. In a retrospective analysis, Huang et al. (2013) also found a 4-fold increased complication rate for malnourished patients undergoing joint replacement surgery. Bohl et al. (2016) reported 2- to 3-fold increased rates for adverse events, such as surgical site infection, pneumonia, and readmission in patients after total joint arthroplasty. In a prospective study, the risk of unplanned ICU admission (corresponding to Clavien–Dindo IV° complications) was found to be 4 times higher for malnourished patients (Kamath et al. 2016). Taken together, the findings of our study are consistent with existing literature and emphasize the importance of malnutrition as a risk factor for adverse events after orthopedic surgery.

Severe vitamin D deficiency led to a 2-fold increase in falls in our study. Furthermore, multivariable analysis identified severe vitamin D deficiency as an independent risk factor for falls in patients undergoing orthopedic surgery. The role of vitamin D in fall prevention is discussed controversially among experts. In a meta-analysis of randomized controlled trials, vitamin D supplementation reduced the risk of falling by 19% (Bischoff-Ferrari et al. 2009). Conversely, in a sequential trial analysis with a risk reduction threshold of 15% conducted by Bolland et al. (2014), the effect on falls lay within the futility boundary. Although statistically not significant, we found an almost 2-fold increased risk of postoperative delirium in patients with severe vitamin D deficiency. Interestingly, recent studies found evidence for a protective role of vitamin D in the occurrence of hospital-acquired delirium (Bowman et al. 2019). In our study, vitamin D deficiency had no impact on rates of reoperation and readmission or surgical and internal complications. In contrast, Hegde et al. (2018) found a correlation between preoperative vitamin D deficiency and reoperation rate as well as rates of different complications (i.e., thrombosis, myocardial infarction, cerebrovascular accident) after total knee arthroplasty. However, the possible coincidence of vitamin deficiency and other variables affecting postoperative outcome (i.e., hypoproteinemia, diabetes mellitus, frailty) was not taken into account in the latter study. Taken together, the results of our study provide little evidence that severe vitamin D deficiency is a risk factor for adverse events after orthopedic surgery. However, the role of vitamin D in the context of orthopedic surgery remains the subject of scientific discourse (Maier et al. 2016, Shin et al. 2017, Hegde et al. 2018).

To our knowledge this is the first study to evaluate a possible correlation between deficiency of vitamin B12 and folate and adverse events after orthopedic surgery. In contrast to hypoproteinemia and severe vitamin D deficiency, deficiency of vitamin B12 and folate had no impact on the risk of adverse events after orthopedic surgery.

This study has several limitations that are consistent with most database studies. Data acquisition was limited to the data available from the hospital information system. Due to the retrospective study design, results are susceptible to selection bias. Furthermore, other parameters with possible influence on postoperative outcome, such as BMI and psychosocial aspects, could not be captured. Despite these limitations, our study demonstrates the relevance of malnutrition in geriatric patients undergoing elective orthopedic surgery. Furthermore, to our knowledge this is the first study to research the effects of vitamin B12 and folate deficiency in orthopedic surgery.

Due to the high rate of malnutrition and its negative effects on postoperative outcome, a systematic screening of geriatric patients undergoing elective orthopedic surgery should be applied. As malnutrition is considered as a modifiable risk factor (Schroer et al. 2019), future research should focus on the effects of preoperative nutritional intervention in a prospective randomized setting.

In conclusion, malnutrition and vitamin deficiency are common among geriatric patients undergoing elective orthopedic surgery. As malnutrition and severe vitamin D deficiency represent independent risk factors for postoperative complications, a systematic screening of at-risk patients should be applied.

## Supplementary Material

Supplemental Material

## Supplementary data

Figure 1 is available as supplementary data in the online version of this article, http://dx.doi.org/10.1080/17453674.2021.1882092
