# Supplementary figures and images for: Impact of malnutrition and vitamin deficiency in geriatric patients undergoing orthopedic surgery
Source: Acta Orthop. 2021 Feb 4;92(3):358–63. doi: 10.1080/17453674.2021.1882092 (PMC8231356; doi:10.1080/17453674.2021.1882092)

Supplementary data

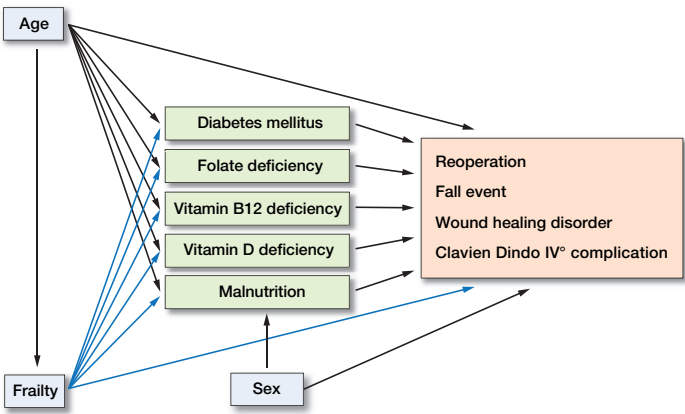

Figure 1. Assumed cause-effect interactions.

Supplement: Supplemental Material [file IORT_A_1882092_SM2372.pdf]
